# Utility of point-of-care ultrasound of the liver in the neonatal intensive care unit: experience from a case series

**DOI:** 10.3389/fped.2025.1632908

**Published:** 2025-08-29

**Authors:** Arpit Sohane, Sujata Deshpande, Rema Nagpal, Yogen Singh, Pradeep Suryawanshi

**Affiliations:** ^1^ Department of Neonatology, Bharati Vidyapeeth Deemed University Medical College and Hospital, Pune, Maharashtra, India; ^2^ Department of Pediatrics, Division of Neonatology, University of California, UC Davis Children’s Hospital, Sacramento, CA, United States

**Keywords:** point-of-care ultrasound, liver, neonate, POCUS, neonatal intensive care

## Abstract

**Introduction:**

Point-of-care ultrasound (POCUS) is increasingly being utilized for the management of sick and premature neonates in the neonatal intensive care unit (NICU). While neonatologist-performed cranial, lung, and cardiac ultrasound have become the standard of care in many NICUs across the world, the use of POCUS for the evaluation of the liver remains relatively unexplored.

**Materials and methods:**

This case series included neonates admitted to a tertiary-care NICU between January 2021 and April 2025, with radiological liver abnormalities detected on point-of-care ultrasound by treating neonatal physicians. Cases were identified from stored ultrasound images, and clinical data were extracted from electronic and physical medical records.

**Results:**

Six patients were identified, in whom a liver POCUS performed by the treating physician revealed a radiological liver abnormality for presenting symptoms that did not include cholestasis or deranged liver function tests. The conditions diagnosed included liver hemangioma, total anomalous pulmonary venous connection, liver hematoma, hepatic abscess, hepatic total parenteral nutrition extravasation, and portal vein thrombosis.

**Conclusion:**

This case series demonstrates the significant role of liver POCUS for the diagnosis of various conditions in neonates, which may be hematological, cardiac, infectious, hemorrhagic, or iatrogenic in origin. Incorporation of liver POCUS into NICU protocols for screening symptomatic infants, particularly those with umbilical venous catheters, pulmonary hypertension, congestive cardiac failure, or unexplained deterioration, may expedite early diagnosis and management of such conditions.

## Introduction

Point-of-care ultrasound (POCUS) is ultrasonography performed and interpreted by the clinician at the bedside, allowing a direct correlation with clinical presentation (1). It expedites clinical diagnosis, while providing an advantage of repeatability and longitudinal monitoring of a sick patient (2).

POCUS is increasingly being utilized in the neonatal intensive care unit (NICU) for the management of sick and premature neonates (3). Some of the common applications include functional echocardiography (FnECHO) for the assessment of hemodynamics, lung ultrasound for the diagnosis of respiratory conditions, cranial ultrasound (CUS) for the screening for intraventricular hemorrhage (IVH), and sonographic guidance for procedures (2, 4–6).

Terminologies such as “Neonatologist-Performed Echocardiography” (NPE) and “Neonatologist-Performed Lung Ultrasound” (NPLUS) are increasingly recognized worldwide to distinguish ultrasound examinations conducted by neonatologists from those performed by cardiologists or radiologists (7, 8). Cranial, lung, and cardiac ultrasounds conducted by neonatologists have now become routine in many NICUs across the world (9), with numerous academic institutions offering formal training courses in these fields (10, 11). Abdominal ultrasound is gaining recognition for the evaluation of bowel pathologies such as necrotizing enterocolitis (12, 13). Recently, Elsayed and Soylu have described a stepwise evaluation of the abdominal organs in neonates and children using POCUS (14). However, abdominal and hepatic ultrasounds are currently not a part of most standard neonatal POCUS training programs and largely come under the realm of the radiologist for indications such as neonatal cholestasis (15). Neonatologist-performed POCUS to screen the liver is therefore less frequently utilized and is perhaps a relatively unexplored tool in the management of sick patients in the NICU.

In this study, we describe a case series of six neonates in whom point-of-care liver ultrasound performed by the treating neonatal physician aided in the diagnosis of a variety of neonatal conditions, leading to early targeted intervention.

## Materials and methods

This case series included neonates admitted to the tertiary-care NICU of a university hospital, who had radiological liver abnormalities detected by bedside point-of-care ultrasound performed by neonatal physicians, for presenting symptoms that did not include cholestasis or deranged liver function tests. Cases were identified by reviewing ultrasound images stored during the period between January 2021 and April 2025 in the unit's point-of-care imaging software. Additional relevant details, such as maternal and birth history, clinical course during hospital stay, indications for performing liver POCUS, management and outcome, were obtained from electronic and physical medical records. Patient identifiers were kept confidential. Institutional ethics committee approval was obtained for reporting this series (Ref: BVDUMC/IEC/110E/25-26). The AME Case Series Checklist (adapted from CARE and PROCESS Checklists) was used to report this case series (16).

## Results

We identified six patients in whom a liver POCUS performed by the treating physician revealed a radiological liver abnormality for presenting symptoms that did not include cholestasis or deranged liver function tests. The details of presentation, management, and outcomes are described under “Case Descriptions”. The conditions diagnosed included liver hemangioma, total anomalous pulmonary venous connection (TAPVC), liver hematoma, hepatic abscess, hepatic TPN extravasation, and portal vein thrombosis (PVT). A summary of the presenting features and radiological diagnoses of the patients is also depicted in Table 1.

**Table 1:** Clinical and radiologic characteristics of the study neonates.

Case number	Gestational age	Birth weight	Presenting symptoms (day of life)	Indication for performing liver POCUS	Findings of liver ultrasound
1	38 + 3 weeks	2,500 g	Respiratory distress and clinical features of congestive cardiac failure (day 2)	Incidentally found liver mass on subcostal view during FnECHO	Hemangioma of the liver
2	36 + 4 weeks	1,600 g	Progressive respiratory distress and oxygen requirement on CPAP (day 6)	Incidentally found liver mass on subcostal view during FnECHO	Saccular hypoechogenicity: venous confluence of infradiaphragmatic TAPVC
3	27 + 4 weeks	970 g	Pallor and sudden drop in hemoglobin (day 6)	To look for internal organ hemorrhage	Liver hematoma
4	31 + 4 weeks	1,460 g	Abdominal distension, bilious vomiting, fever (day 6)	Clinical deterioration, unexplained hepatomegaly	Liver abscess
5	27 + 5 weeks	1,005 g	Inability to wean NIV support (day 17)	Incidentally found heteroechoic mass in the liver while performing a lung ultrasound	Hepatic TPN extravasation/hematoma
6	27 + 3 weeks	1,120 g	Persistent thrombocytopenia (day 9)	To screen for thrombosis in internal organs	Portal vein thrombosis

## Case descriptions

### Case 1

A female neonate, weighing 2.5 kg, was born to a gravida 3 para 2 mother at 38 + 3 weeks gestational age (GA), by caesarean section for fetal distress and meconium-stained amniotic fluid (MSAF). After a trial of non-invasive (NIV) respiratory support, the infant was referred to our tertiary care center for persistent respiratory distress at 45 h of life (HOL).

On admission, the neonate was normothermic, with an oxygen saturation of 85% on room air; had tachycardia and tachypnea, with subcostal, intercostal recessions. There were basal crepitations, a gallop rhythm on auscultation, and hepatosplenomegaly. NIV support was commenced. Diuretic and dobutamine were started for clinically suspected congestive cardiac failure (CCF). Blood investigations showed a hemoglobin level of 123 gm/L; C-reactive protein (CRP) 69.5 mg/L (normal <5 mg/L); thrombocytopenia (platelet count 40 × 10^9^/L). Antibiotics were administered due to raised CRP and suspected sepsis.

**Figure 1:**
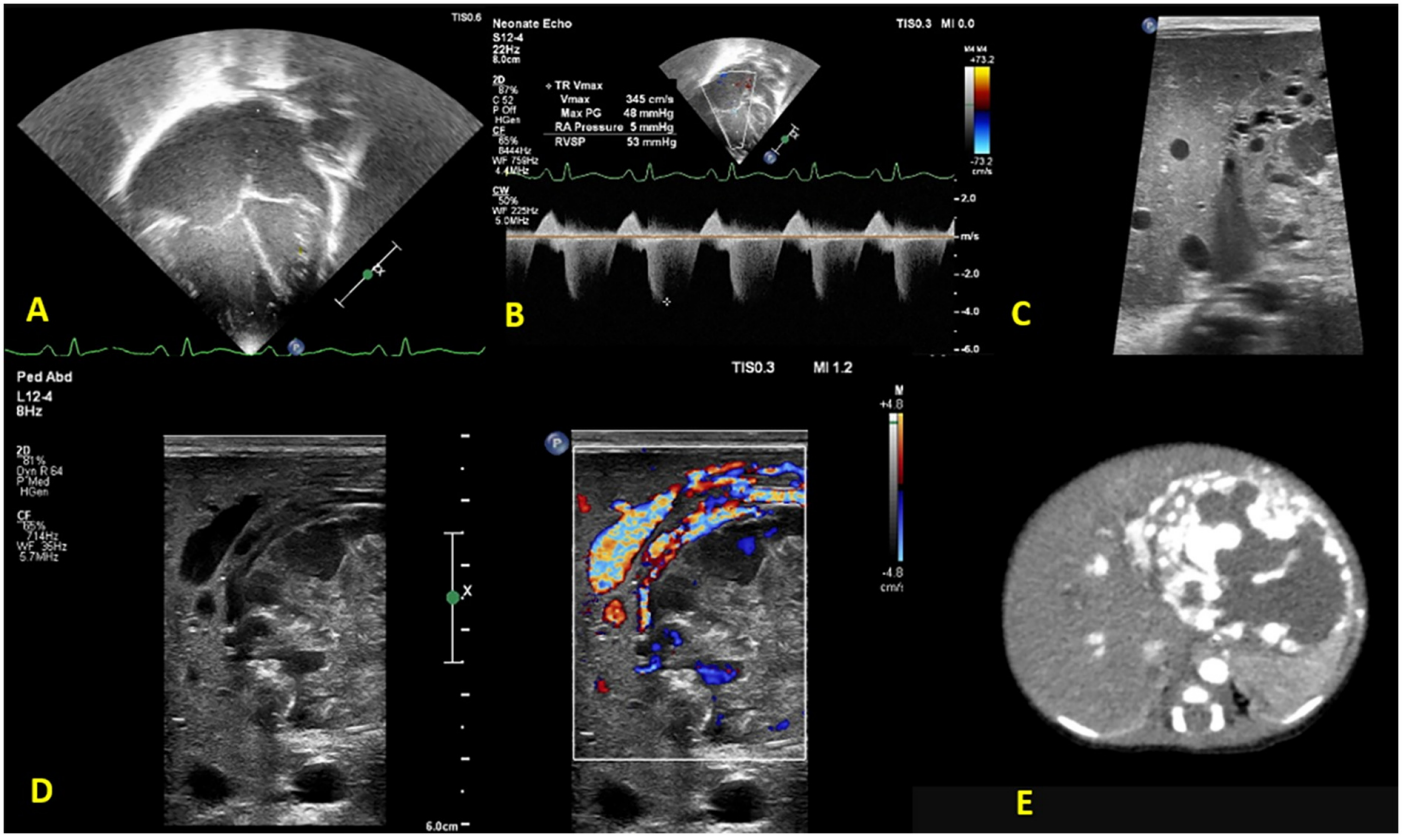
Functional echocardiography shows **(A)** a dilated right atrium and right ventricle **(B)** tricuspid regurgitation with pulmonary arterial systolic pressure 50–55 mmHg. Liver POCUS reveals **(C)** a non-homogeneous mass, measuring 5 × 5 cm in the left lobe of the liver with **(D)** excessive vascularity on Doppler evaluation, suggestive of a liver hemangioma. **(E)** Confirmatory CT abdomen with contrast.

After initial stabilization, a bedside FnECHO was performed, which revealed a dilated right atrium and right ventricle, a high right ventricular output and normal left ventricular output, normal cardiac systolic functions, features of pulmonary hypertension (PH) [pulmonary arterial systolic pressure (PASP) by tricuspid regurgitation (TR) jet 50–55 mmHg] (Figures 1A,B), a D-shaped left ventricle, and a right to left shunting across the patent foramen ovale (PFO). The subcostal echocardiographic views showed a significantly dilated inferior vena cava (IVC) and left hepatic artery. A further interrogation of the liver revealed a non-homogeneous mass measuring approximately 5 × 5 cm in the left lobe of the liver, with excessive vascularity on Doppler evaluation, suggestive of a liver hemangioma (Figures 1C,D). The ultrasound findings were confirmed by the radiologist, and a subsequent CT abdomen revealed a well-defined oval lesion measuring 6 × 5 × 4.8 cm in segments II and III of the left lobe of the liver, suggestive of high-flow hemangioma, confirming the diagnosis (Figure 1E).

Over the following 24 h, the neonate required invasive ventilation for increased respiratory distress and oxygen requirement. There was fresh bleeding from the endotracheal (ET) tube and the coagulation profile was deranged. Fresh frozen plasma (FFP) was transfused, and patient was put on high-frequency oscillatory ventilator (HFOV) support. Pediatric hemato-oncology consultation was done for a likely Kasabach–Meritt phenomenon [D-Dimer 15.76 mg/L (reference range up to 0.243 mg/L)]; IV methyl prednisolone was started, with a plan to start propranolol after hemodynamic stability. A pediatric surgery consult suggested left lobe hepatectomy after stabilization.

Because the neonate remained hemodynamically unstable, a trans-atrial coil embolization of left hepatic artery branches through the left brachial artery was performed by the interventional radiologist. There was transient improvement in pulmonary hypertension parameters; however, thrombocytopenia and coagulopathy persisted, requiring multiple transfusions of platelets and plasma. On DOL 9, the patient had an episode of endotracheal bleeding, with rapid deterioration thereafter; and eventually succumbed following a massive pulmonary hemorrhage.

### Case 2

A late-preterm infant (GA 36 + 4 weeks), weighing 1,600 g, was born to a primigravida mother, via spontaneous vaginal delivery and MSAF. The infant developed respiratory distress soon after birth and was referred to our hospital on DOL 6 for decreased activity, delayed perfusion, and worsening respiratory distress.

**Figure 2:**
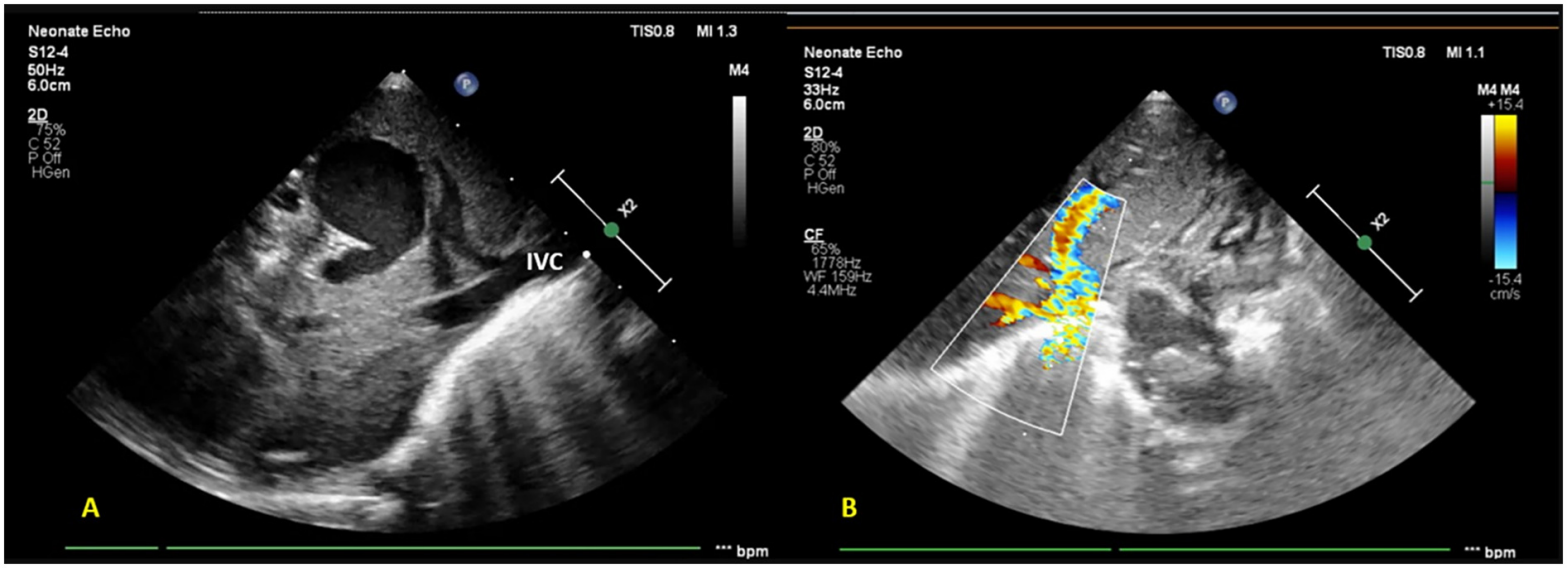
Subcostal imaging and targeted liver POCUS views reveal **(A)** a saccular hypo-echogenicity in the liver (likely a venous confluence draining into the portal tract) further draining into the inferior vena cava **(B)** color Doppler showing an anomalous vessel with turbulent flow.

On admission, the infant was normothermic, with tachycardia, tachypnea, and subcostal and intercostal retractions; oxygen saturation (SpO2) was 88% on room air. Continuous positive airway pressure (CPAP) support with binasal prongs was commenced, with an oxygen requirement of 30% to maintain SpO2 of >90%. No pre-post ductal saturation difference was noted. A provisional diagnosis of meconium aspiration syndrome (MAS) with suspected sepsis was made. Blood investigations revealed a normal hemogram and CRP. A chest radiograph was suggestive of bilateral non-specific air space opacities. A routine screening FnECHO revealed features of mild-to-moderate pulmonary hypertension with PASP ≈45 mmHg, small left atrium (LA), right to left flow across the PFO, normal cardiac outputs, and cardiac systolic function. A subcostal echocardiographic imaging unexpectedly revealed a saccular hypo-echogenicity in the liver. Doppler ultrasound confirmed its vascular nature. On further interrogation, all pulmonary veins were noted to be draining into a chamber confluence and then descending into a large vessel joining the IVC (Figure 2).

The findings suggested an infra-diaphragmatic obstructed TAPVC with moderate pulmonary hypertension, which was confirmed by the pediatric cardiologist. The infant was referred to a cardiac surgery center for early definitive surgical repair.

### Case 3

**Figure 3:**
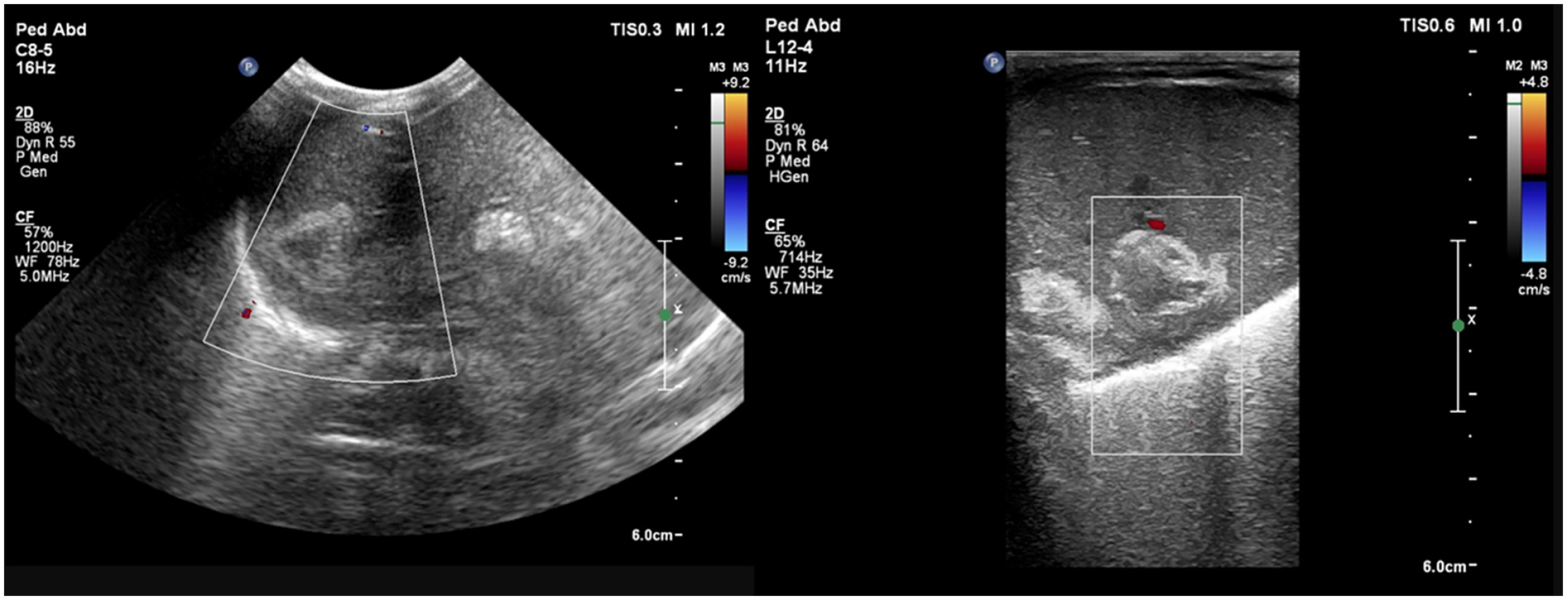
Liver POCUS shows well-defined heterogeneous hypoechoic lesions with a hyperechoic wall and subtle internal septations in the right lobe of the liver. Doppler evaluation shows no internal or perilesional vascularity.

A preterm infant (GA 27 + 4 weeks), weighing 970 g, was born via emergency caesarean section for a premature preterm rupture of membranes since a week. The mother received intrapartum antibiotics and a single dose of steroid prior to delivery. In the NICU, the infant required intubation for significant respiratory distress and two doses of surfactant subsequently. First-line antibiotics were started; the umbilical venous catheter (UVC) and umbilical arterial catheter (UAC) were secured. The initial hemogram and CRP were normal. Minimal enteral nutrition was started on day 2. The patient was extubated to NIV support on DOL 6. On the same day, a clinical pallor was observed, and the complete blood count revealed a drop in hemoglobin levels to 119 gm/L (from 134 gm/L a day prior). There was no hypotension or metabolic acidosis on blood gas. Packed cell transfusion was arranged, and a bedside POCUS screening was performed for concealed hemorrhage. The cranial ultrasound result was normal, while the abdominal ultrasound revealed multiple well-defined heterogeneous hypoechoic lesions with internal septations in the right lobe of the liver, the largest one measuring approximately 3 × 2 cm with a hyperechoic wall. There was no internal or perilesional vascularity on Doppler (Figure 3). The possibilities of liver abscess or liver hematoma were considered; umbilical catheters were removed and antibiotics escalated. However, serial CRPs were negative on DOL 6, 7, and 8, respectively, and blood cultures were sterile. The baby remained clinically stable, achieved full feeds, and antibiotics were stopped on day 10. An abdomen magnetic resonance imaging (MRI) performed on DOL 13 was suggestive of a well-defined cystic lesion in the right lobe of the liver with peripheral hemosiderin deposit, suggestive of subacute or chronic hematoma. Further hospital stay was uneventful, and the baby was discharged home on DOL 54 in a stable condition. Follow-up abdominal ultrasounds (day 18 and day 66) showed a progressive decrease in the size of the lesion.

### Case 4

A preterm male infant (GA 31 + 4 weeks), weighing 1,460 g, was born to a gravida 5 mother, by caesarean section for abnormal fetal Dopplers. The mother was on medication for overt diabetes and chronic hypertension, with a history of one miscarriage and three previously unevaluated intrauterine deaths. She received a full course of antenatal steroids prior to delivery. At birth, the infant required delivery room intubation for resuscitation. In the NICU, the neonate required mechanical ventilation and two doses of surfactant 8 h apart, for significant respiratory distress. Empiric antibiotics were started as per unit protocol. Umbilical venous and arterial lines were secured, and tip positions were confirmed by a plain radiograph. The results of routine blood investigations at 12 h of life were normal. Trophic feeds were started on day 2 and graded up as tolerated. On DOL 4, the baby had two episodes of vomiting associated with mild abdominal distension, following which feeds were temporarily withheld. The neonate was extubated to NIV support on DOL 5 and feeds were restarted (expressed breast milk). Antibiotics were stopped since the results of culture tests were negative. A peripherally inserted central catheter was planned after the removal of the UVC. On DOL 6, the baby developed fever, abdominal distension, and multiple episodes of bilious vomiting. Feeds were withheld; a septic workup showed thrombocytopenia and a deranged coagulation profile. Antibiotics were escalated. An x-ray abdomen showed an enlarged liver and the UVC in the liver at the level of T12. The UVC was removed. The patient showed signs of hemodynamic instability, was electively intubated, and inotropes commenced. Platelets and FFP were transfused.

**Figure 4:**
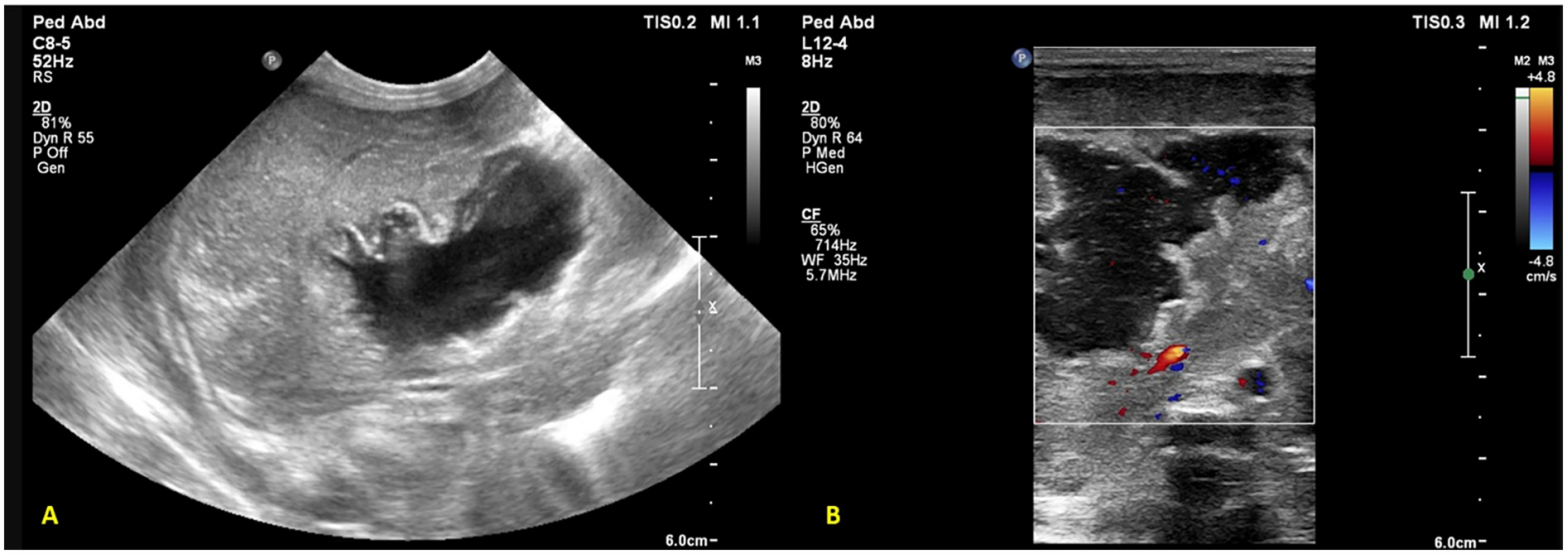
Liver POCUS shows **(A)** a lesion with a thick and hyperechoic wall in the right lobe of the liver measuring 5 × 2.8 × 3.7 cm with **(B)** a cystic center and mobile echoes within the lesion, with no increase in vascularity suggestive of an abscess.

In view of the patient’s clinical deterioration and unexplained hepatomegaly, a screening point-of-care abdominal ultrasound was performed, which showed a thick, hyperechoic walled lesion measuring 5 × 2.8 × 3.7 cm, with a cystic center, with mobile echoes within, and no increase in vascularity, suggestive of an abscess in the right lobe (Figure 4). Other abdominal organs, like spleen and kidneys, did not show any focal lesion. The results of the cranial ultrasound were normal. The findings were confirmed by a radiologist.

Intravenous antibiotics were further escalated, and an ultrasound-guided aspiration/drainage of the abscess was planned but deferred due to the deranged coagulation. The clinical condition deteriorated rapidly in the next 12 h, despite all supportive measures. The patient succumbed following a massive pulmonary hemorrhage on DOL 7. The blood culture subsequently grew *Enterobacter cloacae*, resistant to carbapenems, cephalosporins, and sensitive to amikacin.

### Case 5

**Figure 5:**
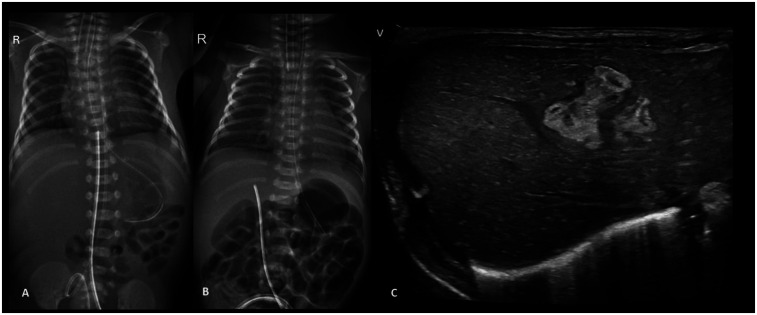
Plain chest and abdomen radiograph showing **(A)** a UVC tip at the level of the diaphragm (T9–10) **(B)** on day 3, the UVC tip shows migration to the T12 level, likely in hepatic parenchyma **(C)** liver POCUS showing a heteroechoic mass in the right lobe of the liver.

An extremely preterm (27 + 5 weeks), VLBW (1,005 g), male neonate was born to a gravida 2 mother via spontaneous vaginal delivery, with a maternal history of PPROM of 9 h. In the NICU, the neonate received mechanical ventilation and surfactant therapy for respiratory distress within one hour of birth. The UAC and UVC were secured. On check x-ray, the UVC tip was found at the level of the diaphragm, T9-T10 (Figure 5A). First-line antibiotics and TPN were initiated. The neonate was extubated at HOL 24 to NIV support. The UAC was removed on day 3. The infant was reintubated at HOL 70 for worsening respiratory distress on NIV support. A FnECHO was suggestive of a hemodynamically significant PDA and paracetamol was started for PDA closure. A postintubation x-ray incidentally revealed a UVC tip in the liver (Figure 5B), which was adjusted to a low-lying position. The neonate was again extubated on DOL 6 and the UVC was removed on DOL 7. On DOL 9, the baby had abdominal distension associated with bilious aspirates; a sepsis workup showed rising levels of CRP and thrombocytopenia; antibiotics were upgraded further. Blood culture grew *Sphingomonas paucimobilis* and antibiotics were adjusted as per the sensitivity report. There was clinical improvement, and the neonate remained hemodynamically stable thereafter. On DOL 17, while performing a lung ultrasound for inability to wean NIV support, the neonatal trainee incidentally found a hyperechoic lesion in the right lobe of the liver, measuring 1.4 cm × 1.0 cm × 1.4 cm (Figure 5C). This was confirmed by the radiologist, and a differential diagnosis of liver hematoma or TPN extravasation was considered. The neonate is currently stable on CPAP and full feeds. A follow-up ultrasound on DOL 34 showed regression in the size of a lesion.

### Case 6

**Figure 6:**
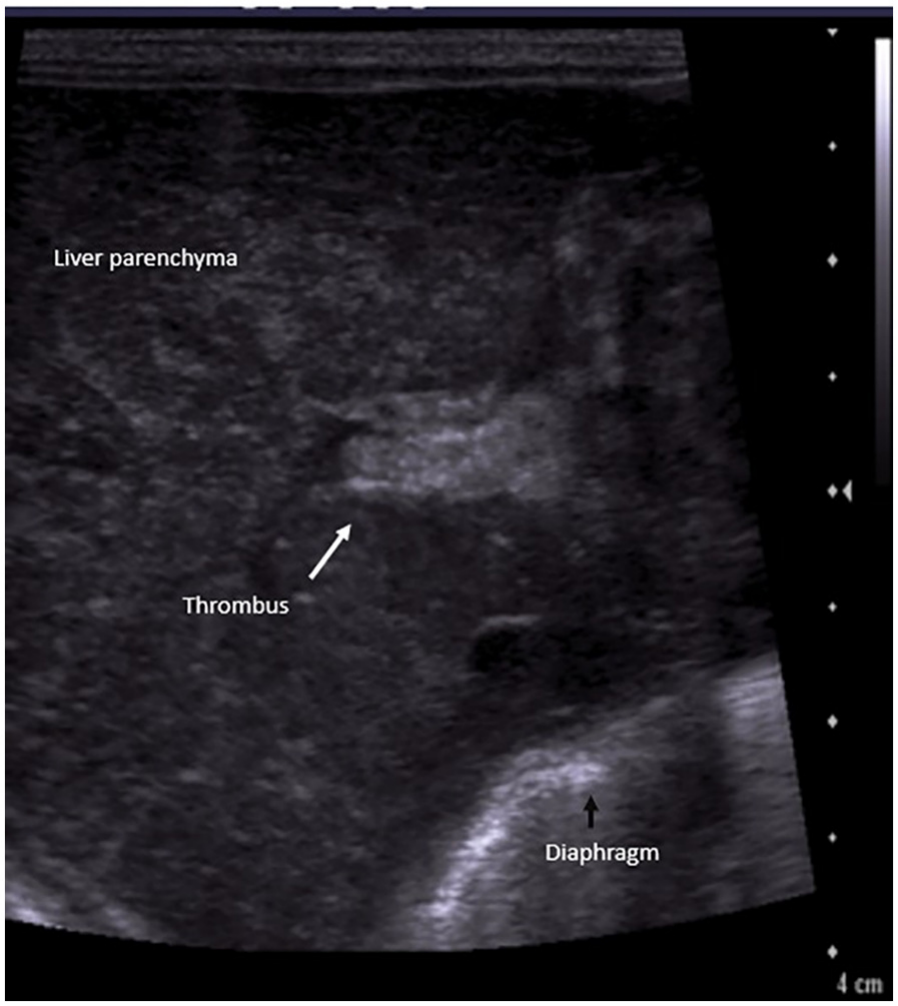
Liver POCUS shows an echogenic thrombus (arrow) in the portal vein.

A preterm (GA 27 + 3 weeks) infant, weighing 1,120 g, was born to a gravida 2 mother via emergency cesarean section in view of antepartum hemorrhage and required delivery room intubation for resuscitation. The baby was transferred to the NICU, where it developed significant respiratory distress requiring mechanical ventilation. A chest x-ray showed a ground glass appearance of the lungs with air bronchograms, and surfactant was administered at 2 h of life. Umbilical venous and arterial catheters were secured and antibiotics commenced. The initial hemogram was normal. A second dose of surfactant was administered for high oxygen requirement, following which ventilator settings were weaned. On DOL 4, the neonate had an episode of pulmonary hemorrhage. Investigations revealed thrombocytopenia and a deranged coagulation profile. Blood products were administered, and the patient was placed on HFOV. Repeat blood cultures and endotracheal secretions grew *Stenotrophomonas maltophilia*, which was sensitive to fluroquinolones and ceftazidime; antibiotics were modified accordingly. The patient improved clinically. However, as thrombocytopenia persisted despite clinical improvement, adequate antibiotic coverage, and platelet transfusions, additional causes for thrombocytopenia were considered. A screening bedside ultrasound of the abdomen to screen for thrombosis in major vessels revealed an echogenic thrombus in the right and left portal vein, with a reformation of the distal portal vein (Figure 6). The umbilical lines were removed, and the antibiotics were continued for a total of 14 days. The patient was subsequently extubated and discharged on DOL 66. A repeat ultrasound abdomen prior to discharge showed normal flow through the portal vein.

## Discussion

Although the six cases described above have distinct diagnoses and underlying pathophysiology, a common feature that stands out is that all of them were detected following a point-of-care ultrasound of the liver by the neonatal physician, either incidentally or on a targeted POCUS of the liver/abdomen. Many consensus guidelines recommend CUS and FnECHO either in preterm infants as a routine screening procedure or in symptomatic patients in the NICU with sudden clinical deterioration (17, 18). The cases described above, however, demonstrate the utility of a *targeted* POCUS of the liver, for the diagnosis of a variety of conditions in a symptomatic neonate.

The first two cases (liver hemangioma and TAPVC) presented with respiratory distress and oxygen requirement and were incidentally diagnosed by the treating neonatal physician due to abnormal masses seen in the liver while performing a FnECHO. The fifth case was diagnosed incidentally while performing an ultrasound of the lung. The remaining cases were diagnosed with targeted POCUS of the liver and abdomen in symptomatic neonates performed for unexplained deterioration. The neonatologists who performed the POCUS scans in these patients have received training by accredited trainers of the Australasian Society for Ultrasound in Medicine (ASUM) and the “Neonatal Hemodynamics and Targeted Neonatal Echocardiography (TNE) Subspecialty Fellowship training program” from the University of Toronto, Canada. The training primarily focused on neonatal cardiac, cranial, and lung ultrasound. Although they also pursued additional hands-on training in abdominal ultrasound, this was not part of the structured program. Most existing neonatal POCUS training programs do not routinely include abdominal and liver POCUS in their curricula, and our case series highlights this gap. In a recent review article, Elsayed and Soylu described a methodical evaluation of the abdominal organs in neonates and children using POCUS, which includes stepwise scanning of the upper and lower abdomen, as well as the kidneys and bladder (14). Similar to the ASUM and TNE programs, a curriculum comprising didactic teaching, supervised hands-on sessions, and image review by a team of neonatologists proficient in abdominal POCUS and of pediatric radiologists would perhaps help trainees in acquiring adequate proficiency.

### Liver hemangioma

Hepatic hemangiomas are benign vascular tumors classified as Congenital Hepatic Hemangiomas (CHHs) and Infantile Hepatic Hemangiomas (IHHs) (19). CHHs develop *in utero*, reaching peak size prior to or at birth. Despite being categorized as benign tumors, the high cardiac output failure in patients with CHH can be challenging to manage and may often prove fatal (20). In addition, patients may present with intratumoral bleeding, anemia, thrombocytopenia, and hypofibrinogenemia soon after birth. IHHs evolve and proliferate until 6–12 months of life and may present with heart failure in the postneonatal period.

On ultrasound, focal hemangiomas usually appear as hypoechoic masses with central necrosis or fibrosis and dilated vessels. CHHs, when compared with IHHs, are well-circumscribed, large lesions that are heterogeneous on both ultrasound and MRI images (15). Color Doppler imaging reveals prominent, high-flow vascular structures (21).

Similar to our case, Zhou et al. (22) and Singh et al. (23) reported neonates who presented with respiratory distress and echocardiographic features of PH. A liver ultrasound performed for hepatomegaly revealed hepatic hemangioma/arteriovenous malformation. The patients showed improvement after coil embolization and diuretic therapy. Unlike our patient, there was no thrombocytopenia or derangement of coagulation parameters. Propranolol is known to cause a regression of lesions in cases of infantile hemangiomas (24). However, the risk vs. benefit needs to be considered in patients with high-output cardiac failure.

Pulmonary hypertension seen in neonates is usually associated with sepsis, asphyxia, cardiac, or pulmonary pathologies. However, extracardiac causes of PH must be explored by screening the POCUS of the brain, liver, and abdomen.

### TAPVC

Infradiaphragmatic TAPVC is a congenital cardiac defect in which the pulmonary veins do not drain into the LA but instead drain via a common vertical venous confluence into the portal venous system, IVC, or ductus venosus. Obstruction to the abnormal pulmonary veins is most commonly seen in the infradiaphragmatic variety (23).

Most case reports of infradiaphragmatic TAPVC in the literature have described neonates presenting with severe respiratory distress and profound cyanosis (26, 27). Echocardiography performed for severe cyanosis not responding to oxygen usually reveals the diagnosis. However, our patient was hemodynamically stable on CPAP, with an oxygen requirement of 30%, and a provisional diagnosis of MAS. A routine FnECHO performed for PPHN at 12 h of admission revealed a mass in the liver in the subcostal view. Further evaluation led to definitive diagnosis.

### Liver hematoma

Umbilical venous catheter placement is a common procedure in the NICU and may be associated with a wide range of complications such as hepatic abscesses, hematomas, lacerations of liver parenchyma, and portal venous thrombosis (28). Nonhepatic complications include pleural effusions, pericardial effusion, cardiac tamponade, and arrythmias (29). Hematomas in the liver can also be associated with birth trauma, chest compressions during resuscitation, sepsis, and prematurity (30). We speculate that the liver hematoma found in our case was secondary to a misplaced UVC.

Cohen et al. described a case of subcapsular hematoma following a UVC placement in a preterm infant. After initial resistance, the UVC tip was kept in a low-lying position. On day 3 of life, the infant developed anemia, hypotension, and abdominal distension with no evidence of IVH on cranial ultrasound. The infant succumbed and postmortem examination revealed the UVC entering the liver through the umbilical vein and a hematoma of the right lobe of the liver (30). A similar case of hepatic hematoma and laceration secondary to a malpositioned UVC was described by Yiğiter et al. in a 30-week preterm neonate who also succumbed due to massive liver hemorrhage and circulatory shock. The diagnosis was made by using a liver ultrasound with a confirmatory CT scan (31).

On ultrasound, parenchymal hemorrhage in the liver appears hyperechogenic, or it may appear cystic with a hyperechogenic wall. The patient's clinical and radiological course may help differentiate it from an abscess (32).

Liver hematomas secondary to UVC misplacement are to be treated with prompt removal of the UVC, followed by active monitoring for natural resolution (33). POCUS is increasingly becoming a gold standard for confirming the position of the catheter tip after UVC placement (34, 35). Our case emphasizes the need for periodic follow-up ultrasound of the liver for the migration of line and malpositioning.

### Liver abscess

Neonatal liver abscess is a rare entity, and a high index of suspicion is required to diagnose abscess in a septic neonate. It can be caused by an ascending infection through the UVC, particularly if malpositioned in the liver parenchyma, or through the blood stream in culture positive sepsis. Other risk factors include omphalitis, prematurity, NEC, abdominal surgery, total parenteral nutrition (TPN) administration through the umbilical catheter, or immunodeficiency disorders (36). Symptoms may be non-specific and liver functions may be normal except for a low serum albumin level. The condition possesses high morbidity and mortality (37).

Similar to our case, Lam et al. described a 27-week preterm infant with RDS, requiring two doses of surfactant (38). A UVC placed on day 1 of life showed the tip to be in the liver shadow on plain x-ray. Parenteral nutrition was infused through the UVC, and on day 9 of life, the patient had abdominal distension. Necrotizing enterocolitis was suspected and the UVC removed. The patient’s condition, however, deteriorated clinically, and the patient developed hypotension, thrombocytopenia, and coagulopathy. An abdominal ultrasound revealed a septated well-circumscribed mass in the liver, which was aspirated by percutaneous needle drainage. Despite antibiotics and aggressive supportive treatment, the patient died 12 h after the procedure. An autopsy revealed a large hepatic abscess with no evidence of NEC. In a case report, Du et al. have described a neonate who presented with fever with no other associated symptoms. A POCUS screening for deep abscess revealed an abscess in the liver (39).

On ultrasound, liver abscesses may be solitary or multiple, and are hypoechoic, avascular, multilocular collections with a hyperechogenic rim. Tumors on the other hand, usually have a solid appearance with calcification and tend to be more heterogeneous due to the presence of necrotic and hemorrhagic areas (15).

As our patient had a UVC *in situ* from day 1, it is likely the abscess was a complication of malpositioning of the UVC tip. A screening POCUS for follow-up of the UVC position, or a high index of suspicion for even minor episodes of feed intolerance, might have detected the abscess earlier, with perhaps a more favorable outcome.

### Hepatic TPN extravasation/hematoma

There are several case reports in the literature describing complications of a malpositioned UVC in neonates, which include hematomas, extravasation injuries, and abscesses of the liver (29, 34, 40). Because our patient was clinically stable on CPAP at the time this lesion was incidentally found on ultrasound, we suspected the liver mass to be hematoma or a TPNoma. While hepatic hematoma may result due to vascular injury by the UVC tip, extravasation of hypertonic and alkaline TPN solutions can lead to tissue necrosis within the liver parenchyma (TPNoma) (41, 42). Clinical symptoms in both hematoma and TPN extravasation may vary from minor symptoms such as abdominal distension and hepatomegaly to severe, acute cardiovascular compromise, associated with pallor and shock. A high index of suspicion in the presence of risk factors (low-lying UVC, prolonged duration of the catheter, extremely preterm/very low-birth-weight infants) and a prompt screening ultrasound of the liver and abdomen may lead to an early diagnosis.

On ultrasound, a TPNoma may appear as a hyperechoic mass in the acute stage. A subacute lesion may appear as an irregular heterogeneous mass, with a hyperechoic rim (TPN lipid) and hypoechoic, cystic, central area of aqueous material or necrosis. Over time, these lesions may regress in size with calcification. Ascites may be associated if there is a peritoneal extravasation of TPN following a disruption of the liver capsule. In a five-year case series of 33 neonates with hepatic extravasation complicated by the UVC, Chen et al. described TPNomas to be characteristically lobulated or wedge shaped (43). Hematomas may also appear hyperechoic in early stages on ultrasound and hypoechoic in later stages, often containing internal septations (44). Serial imaging is needed to follow the evolution. A CT or MRI imaging may help in differentiating a hepatic hematoma (peripheral hemosiderin deposit) from a TPNoma (fat deposits) (44, 45).

Our case highlights the need for follow-up ultrasounds in neonates with a UVC for displacement and removal of the catheter if malpositioned. In a patient with a UVC, a POCUS of the liver and abdomen for unexplained clinical deterioration may lead to early detection of catheter-related complications.

### Portal vein thrombosis

With the use of POCUS in the NICU, PVT is being increasingly recognized in neonates, particularly those with UVC placement. Studies indicate that the incidence of PVT in infants varies from 0% to 43% among neonates with UVCs (46–48).

In acute stages, PVT appears iso/hypoechoic on gray scale ultrasound. Subsequently over the following days, the thrombus appears echogenic, and in late stages, calcification might be seen. Colour Doppler reveals absent or partial blood flow in the portal vein, or the formation of collaterals around the thrombotic segment (40).

In a retrospective study, Solgun et al. evaluated 23 infants with PVT for risk factors and outcomes and found UVC insertion (91.3%) and sepsis (73.9%) to be the leading causative factors for PVT (49). In a retrospective study to describe outcomes of neonates with PVT, Bhatt et al. found that 96% of 74 infants had no complications, and thrombus resolution was documented in 63% of treated and 58% of non-treated infants (50). Careful monitoring and follow-up are needed for long-term complications such as portal hypertension (51, 52).

### Potential pitfalls of liver POCUS

While liver POCUS performed by the neonatologist holds significant promise in the management of sick neonates in the NICU, there are several potential limitations of this imaging modality. Some of these include dependence on the operator's level of skill and training; patient factors, such as bowel gas or ascites, which may affect image quality; and challenges in distinguishing hepatic lesions, such as hemangiomas, from other solid masses. These pitfalls underscore the need for close collaboration with radiologists and advanced scanning when necessary (14, 32).

## Conclusion

This case series demonstrates the utility of POCUS of the liver for detecting a wide variety of conditions that may be hematological, cardiac, infectious, hemorrhagic, or iatrogenic in etiology. While POCUS of the brain and heart is usually advocated in a sick neonate requiring intensive care, ultrasound of the liver is often overlooked as a screening procedure. We recommend that a bedside liver POCUS should be performed in sick neonates in the NICU, particularly those presenting with clinical features of pulmonary hypertension, congestive cardiac failure, and sudden unexplained deterioration. In patients with UVC placement, periodic follow-up ultrasound of the liver should be performed for migration of the line and misplacements. We recommend the incorporation of liver POCUS in NICU protocols to enhance day-to-day management of sick neonates in the NICU, as well as the inclusion of abdominal and liver ultrasound in standard neonatal POCUS training programs.

## Data Availability

The original contributions presented in the study are included in the article/Supplementary Material, and further inquiries can be directed to the corresponding author.
